# Response of Sugarcane Rhizosphere Bacterial Community to Drought Stress

**DOI:** 10.3389/fmicb.2021.716196

**Published:** 2021-10-06

**Authors:** Qi Liu, Xiaowen Zhao, Yue Liu, Sasa Xie, Yuanjun Xing, Jicao Dao, Beilei Wei, Yunchang Peng, Weixing Duan, Ziting Wang

**Affiliations:** ^1^Guangxi Key Laboratory of Sugarcane Biology, Nanning, China; ^2^College of Agronomy, Guangxi University, Nanning, China; ^3^State Key Laboratory for Conservation and Utilization of Subtropical Agro-Bioresources, Guangxi University, Nanning, China; ^4^College of Agriculture, Guangxi University, Nanning, China; ^5^Sugarcane Research Institute, Guangxi Academy of Agricultural Sciences, Nanning, China

**Keywords:** core functional bacteria, drought response, rhizosphere bacterial community, drought stress, sugarcane

## Abstract

Sugarcane is an important sugar and energy crop, and its yield is greatly affected by drought. Although a large number of studies have shown that rhizosphere microorganisms can help improve the adaptability of plants to biotic or abiotic stresses, there is a lack of studies on the adaptability of sugarcane rhizosphere microbial communities to host plants. Therefore, we conducted drought stress treatment and normal irrigation treatment on three sugarcane varieties GT21, GT31, and GT42 widely cultivated in Guangxi. Using 16S rDNA sequencing technology to analyze the changes in abundance of the sugarcane rhizosphere bacterial community under different treatments, combined with the determination of soil enzyme activity, soil nutrient content, and sugarcane physiological characteristics, we explored the sugarcane rhizosphere bacterial community response to drought stress. In addition, we used the structural equation model to verify the response path of sugarcane rhizosphere bacteria. The results show that the bacterial community structure in the rhizosphere of sugarcane is stable under normal water conditions. The change in the bacterial community structure under drought stress has a 25.2% correlation with the drought adaptability of sugarcane, but the correlation with drought stress is as high as 42.17%. The changes in abundance of rhizosphere bacteria under drought stress are mainly concentrated in the phylum Rhizobiales and Streptomycetales. This change is directly related to the physiological state of the host plant under drought stress, soil available phosphorus, soil urease and soil acid protease. We investigated the response species of rhizosphere microorganisms and their response pathways under drought stress, providing a scientific basis for rhizosphere microorganisms to assist host plants to improve drought adaptability.

## Introduction

Drought is an abiotic stress affecting agricultural production, resulting in billions of dollars of annual losses worldwide (Lesk et al., 2016). Over the course of evolution, plants have improved their adaptability to drought stress through various mechanisms such as morphological adaptation, physiological adaptation, and cellular regulation (Sahebi et al., 2018). Under drought stress conditions, rhizosphere microorganisms provide a buffer to enhance the drought tolerance ability of plants (Berg et al., 2014). They participate in soil nutrient cycling, organic decomposition, plant hormone secretion promotion, and other processes, which have positive effects on plant health and growth and help in regulating plant adaptability to drought stress (Evelin et al., 2009). The use of microorganisms to increase plant resistance to environmental stress conditions is an emerging ecological strategy in agriculture. Therefore, understanding the role of rhizosphere microorganisms under drought stress and their assembly according to their functions can maximize the power of microbial communities to support plant resistance to drought (Padmanabhan and Hemaprabha, 2018; Singh et al., 2019).

Sugarcane is an important energy crop that provides 60% of the raw material for the world’s sugar and ethanol production (Rocha et al., 2007). It is primarily grown in tropical and subtropical regions, which frequently experience drought stress events, resulting in yield losses of up to 50% (Li et al., 2016). Drought stress delays the elongation of sugarcane leaves, reduces the photosynthetic rate and chlorophyll content of leaves, reduces the absorption capacity of N, and affects other processes, ultimately leading to a decrease in sugar yield (Garcia et al., 2018; Xu et al., 2018). In response to water scarcity, plants have developed a variety of complex resistance and adaptation mechanisms, including physiological and biochemical responses, which vary between and within species (Seleiman et al., 2021). Zhao et al. (2020) reported differences in the drought tolerance ability of sugarcane among different genotypes. Previous studies have shown that sugarcane growth is affected by a variety of rhizosphere microorganisms such as actinomycetes and bacillus (Liu et al., 2020). However, most of those studies focused on fertilization, breeding, transgenesis, and other aspects of sugarcane, with limited information on sugarcane-related microbial responses to drought (Santos et al., 2017; Júnior M. et al., 2019).

Current studies indicate that soil pH, soil enzyme activity, soil C/N content, and salinity have significant effects on microbial community composition, and that microbial communities are more sensitive to soil environmental changes than to other environmental conditions such as climate change and geographical heterogeneity (Fiere and Jackson, 2006; Zhang et al., 2007). Although drought stress influences soil structure and decreases organic matter and available nutrients, the presence of rhizosphere microorganisms can balance soil organic matter content through mineralization (Lin et al., 2019). The changes in community structure can regulate the interaction between nutrition and defense, thereby affecting the conversion of phosphate and other components in the soil to increase the available nutrient content (Castrillo et al., 2017). The members of the microbial community whose abundance changes during the stress period may partly contribute to the plant’s adaptability to stress (Dai et al., 2019). Therefore, it is necessary to explore the primary response flora of root microbes under drought stress, as well as the correlation between the changes in the primary core responsive flora and the plant and soil environment, to fully understand how rhizosphere microbes aid plants in improving their adaptability to adversity.

In this study, we conducted 16S rDNA deep sequencing analysis of the rhizosphere bacterial communities of three sugarcane cultivars under water and drought treatments to resolve the following enigmas: 1. the main effects of drought stress on sugarcane rhizosphere bacteria, 2. differences in the composition of drought-resistant bacterial communities in the rhizosphere of different sugarcane cultivars, and 3. the response mechanism of sugarcane rhizosphere core drought-resistant bacteria to drought stress. Exploring the mechanism underlying the changes in sugarcane rhizosphere bacterial community under drought stress can help in fully understanding the physiological mechanisms of sugarcane rhizosphere bacterial resistance. This will establish a theoretical basis for understanding the assembly process of host plants to rhizosphere microorganisms under drought stress and enable the development of protocols for the application of rhizosphere bacteria to improve drought stress resistance in sugarcane.

## Materials and Methods

### Cultivar Selection and Field Experimental Design

The experiments were conducted in a glass greenhouse at Guangxi University in Nanning, Guangxi, China (107°31′–108°06′E, 22°17′–22°57′N; 83 m a.s.l.) during the summer of 2018. The experimental site is located in an area with a subtropical monsoon climate with long summers and short winters. The average temperature, annual sunshine time, and annual precipitation at the experimental site were 22°C, approximately 2,600 h, and 1,050–1,300 mm, respectively. Soil used for planting was collected from a field that has been under long-term sugarcane cultivation and has the following characteristics: pH, 6.15; organic matter content, 19.47 g/kg; total nitrogen content, 100.5 g/kg; total phosphorus content, 22.4 g/kg; total potassium content, 7.11 g/kg; alkaline hydrolyzed nitrogen, 136 mg/kg; available phosphorus, 83 mg/kg; and available potassium, 77.1 mg/kg. Three sugarcane cultivars, Guitang21 (GT21), Guitang31 (GT31), and Guitang42 (GT42), of the “Guitang” series recommended by the Guangxi Academy of Agricultural Sciences were selected for the experiment. GT21 is a high-yielding and high-sugar-containing sugarcane variety bred by crossing Ganzhe75-65 as the female parent and Yacheng71-374 as the male parent (Huang et al., 2006). GT31 and GT42 were both obtained through the “five-nursery system;” the former is a cross between Yuetang85-177 as the female parent and CP81-1254 as the male parent (Huan-Guang et al., 2011), and the latter is a cross between ROC22 as the female parent and Guitang92-66 as the male parent (Wang et al., 2015). The three sugarcane varieties are widely planted in Guangxi, China, and have strong adaptability to the soil conditions and climate conditions in Guangxi (Huang et al., 2006; Huan-Guang et al., 2011; Wang et al., 2016). Sugarcane cultivars were planted in plastic pots (35 cm upper diameter, 25 cm lower diameter, 35 cm heights) with three drainage holes approximately 1 cm diameter drilled at the bottom. The pots were placed in a glass greenhouse; the average temperature in the greenhouse was 22°C, consistent with the outside temperature, and the plants were illuminated with only natural light.

A total of 60 pots were planted with sugarcane including 20 pots per cultivar, and on average two to three sugarcane buds in each pot. At the seedling stage (80% of the sugarcane buds have 2–3 true leaves), 10 flowerpots of each cultivar were randomly selected for drought treatment (D), and the remaining 10 were control group (C). Each flowerpot was filled with 2 kg soil collected from the top 0–20 cm layer in the sugarcane-planting field. A TDR-100 (Spectrum Technologies, Inc., Aurora, IL, United States) soil moisture rapid test was used to measure the soil water content at a depth of 5–20 cm. Before the seedling period, the sugarcane buds was irrigated every other day based on the measured water demand (Supplementary Figure 1). After the seedling period, the control group was still irrigated every other day. For the drought treatment, the irrigation was completely stopped from the beginning of the seedling period to the end of the experiment.

### Soil Sampling and Determination of Physicochemical Soil Properties

Four weeks after the drought stress began, we collected rhizosphere soil samples of the three sugarcane cultivars under different water treatments. Sugarcane seedlings were dug out carefully by removing soil within an area of 20 cm^2^ around the seedling. The seedlings were shaken violently to remove large soil particles, and the soil attached to the root surface was collected with a brush and sifted through a 2 mm sieve (Pisa et al., 2011). For each cultivar–water treatment combination (GT21C, GT21D, GT31C, GT31D, GT42C, and GT42D), three biological replicates were taken, and a total of 18 soil samples were obtained. Each soil sample was divided into two subsamples, one stored at 4°C and used to measure the physicochemical properties of the soil, and the other was stored at −80°C for the extraction of rhizosphere microbial DNA within 24 h (Zhao et al., 2020).

Chemical properties of the soil were determined as previously reported (Bao, 2000). Carbon content (SOC) was determined *via* the potassium dichromate sulfuric acid oxidation method, nitrogen content (TN) was measured through the semi-micro Kelvin method, and phosphorus content (AP) was determined *via* molybdenum antimony colorimetry. Measurements were conducted using soil sampled from a depth of 10–15 cm. Leaf water potential was measured using a dew point water potential meter (WP4; Decagon Devices, Inc., Pullman, WA, United States). Measurements were performed between 10:00 and 11:00 on the youngest fully expanded leaves (Bai et al., 2012). Soil urease (S-UE), soil acid phosphatase (S-ACP), soil acid protease (S-ACPT), and soil catalase (S-CAT) levels were determined using a kit purchased from Solebao Company (Beijing, China) according to the manufacturer’s instructions. Chlorophyll fluorescence was measured using a portable fluorometer (PAM-210; Walz, Germany). A measuring beam was applied to the leaf of a plant that was dark-adapted for 30 min to measure minimum fluorescence (*F*_0_); this was followed by the application of a saturating pulse to determine the maximum fluorescence (*F*_m_). The *F*_v_/*F*_m_ value was then calculated, where *F*_v_ = *F*_m_ − *F*_0_ (Sharma et al., 2020). The levels of malondialdehyde (MDA) and proline (Pro) were determined according to the instructions provided for the micro determination kits (MDA-2-Y and PRO-1-Y, respectively; Komin, Suzhou, China) (Wang et al., 2019).

### DNA Extraction, Amplification, and Sequencing

Soil DNA was extracted using an E.Z.N.A.^®^ Soil DNA Kit (Omega Bio-Tek, Inc., Norcross, GA, United States) according to the manufacturer’s instructions. Microbial DNA was extracted from 1 g of fresh soil, and extraction was performed three times for each soil sample. The concentration and quality of DNA samples were measured using a NanoDrop One spectrophotometer (Thermo Fisher Scientific, Waltham, MA, United States). Primer pairs 341F (5′-CCTACGGGNGGCWGCAG-3′) and primer 805R (5′-GACTACHVGGGTATCTAATCC-3′) targeting the V3–V4 hypervariable regions of the bacterial 16S rDNA gene were used for PCR (Peiffer et al., 2013). The reverse primer contained a 12 bp error-correcting barcode unique to each sample. Primers were synthesized by Invitrogen (Carlsbad, CA, United States). PCR reactions, containing 25 μL of 2 × Premix Taq (Takara Biotechnology, Dalian Co., Ltd., Dalian, China), 1 μL of each primer (10 M), and 3 μL DNA (20 ng/μL) template in a total volume of 50 μL, were amplified in a thermocycler (BioRad S1000; Bio-Rad Laboratories, Inc., Foster City, CA, United States) as follows: initialization at 94°C for 5 min; 30 cycles of denaturation at 94°C for 30 s, annealing at 52°C for 30 s, and extension at 72°C for 30 s; followed by a 10 min final elongation at 72°C. PCR products were sequenced by Magigene Technology (Guangzhou, China) using the Illumina HiSeq 2500 platform.

Quality filtering of the paired-end raw reads was performed under specific conditions to obtain high-quality clean reads following the Trimmomatic (V0.33, ^1^) quality-controlled process. Overlapping paired-end clean reads were merged using FLASH (V1.2.11, ^2^); spliced sequences of a minimum of 10 overlapped reads generated from the opposite end of the same DNA fragment and with the maximum allowable error ratio of the overlap region of 0.1 were identified as raw tags (Caporaso et al., 2010). Sequences were assigned to each sample based on their unique barcodes and primers using Mothur (V1.35.1, ^3^), following which the barcodes and primers were removed to obtain effective clean tags. Usearch (V10, ^4^) was used to filter and eliminate noise from the data by clustering similar sequences with <3% dissimilarity. Operational taxonomic units (OTUs) of 16S rDNA were selected from the combined reads of clustered OTUs with 97% similarity with the Quantitative Insights Into Microbial Ecology pipeline (VirtualBox Version 1.1.0) (Edgar, 2010). The 16S rDNA gene sequences obtained in this study are deposited in the National Center for Biotechnology Information Sequence Read Archive database ^5^ under the accession number PRJNA655948.

### Statistical and Bioinformatics Analysis

Alpha diversity was estimated using the Chao1 diversity index and Shannon diversity index. Correlations between α-diversity and soil properties were determined using the “corrplot” package in R v. 3.6.3 (Wei et al., 2017). The β-diversity and phylogenetic community comparisons were estimated using weighted and unweighted UniFrac distance matrices. The Mantel test was used to study the relationship between β-diversity and environmental factors and between the enriched bacterial OTUs and environmental factors. Taxonomic composition was determined based on the relative abundances of the dominant phyla. The changes in the relative abundance of bacterial communities in each compartment were evaluated using the “alluvial” and “ggplot” packages in R v. 3.6 (Wickham, 2007). Distance-based redundancy analysis (dbRDA) was used to evaluate the relationships between soil characteristics and soil bacterial OTUs. Mantel tests, principal coordinate analysis, and dbRDA were performed using the “vegan” package in R v. 3.6 (Oksanen et al., 2013).

For all networks, we utilized the “trimmedmeans of M” (TMM) normalized “counts per milli” (CPM) values, conducted Spearman rank correlations between OTUs, and visualized positive and significant correlations (ρ > 0.7, *P* < 0.001). The descriptive and topological network properties were calculated using graphs. Subsequently, a meta-network was constructed to visualize the correlations between different bacterial species in the sugarcane rhizosphere soil. The TMM-normalized CPM values of bacteria were combined to separate OTU tables for the soil and root communities. The abovementioned network properties were calculated, and the community structure within the soil and root meta-networks was explored by identifying network modules. Network modules are substructures of nodes with a higher density of edges within groups than between them. For this, we utilized the greedy optimization of the modularity algorithm (Clauset et al., 2004) as implemented in the graph. Microbial taxa that frequently co-occur with other taxa in microbial co-occurrence networks are considered ecologically important and potentially play a key role within the microbiome (Agler et al., 2016; Ga et al., 2016). Keystone OTUs were identified separately for the soil and root meta-networks and defined as nodes within the top 1% of nodal degree values in each network. This simple definition was prioritized over a more complex method (e.g., based on high degree and low betweenness centrality) because both definitions uncovered largely the same sets of keystone OTUs (Hartman et al., 2018).

### Structural Equation Model

Two pathways were used to measure the direct or indirect effects of drought tolerance, drought stress, and the rhizosphere environment on sugarcane rhizosphere bacterial communities. In path analysis, a structural equation model (SEM) was designed to characterize the variables and assume causality among these variables in the path diagram (Fanin and Bertrand, 2016). In this study, we hypothesized that sugarcane adaptability to drought stress is caused by the drought tolerance of sugarcane varieties and auxiliary regulation of rhizosphere bacteria (Ullah et al., 2019). Soil nutrients and enzyme activities are commonly used to evaluate soil health status, and we used them as variables to reflect the physicochemical soil properties in a soil moisture-deficient environment (Cardoso et al., 2013). Considering the differences in plant adaptability to drought, we used the physiological index of drought resistance in a broad sense to represent the response factors of sugarcane to drought stress in SEM (Khonghintaisong et al., 2018).

Structural equation model was used to evaluate the adequacy of the model using the χ^2^ test (*P* > 0.05) and the approximate root mean square error (RMSEA) (VALUES < 0.05). These statistical tests were performed in R using the “lavaan” package (Gana and Broc, 2019).

## Results

### Effects of Drought Stress on α- and β-Diversity of Sugarcane Rhizosphere Bacteria

**FIGURE 1:**
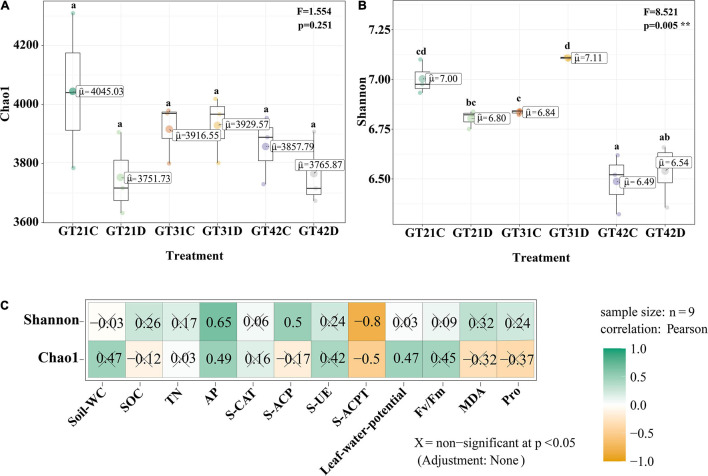
Alpha diversity of rhizosphere microbial community. **(A)** Chao 1 index of α-diversity showed the species richness of rhizosphere microbial community among different treatments. **(B)** α-diversity Shannon index showed rhizosphere microbial community diversity among different treatments. Different letters in the figure represent significant differences between treatments (*P* < 0.05). GT21, GT31, and GT42 are sugarcane varieties, C is normal watering treatment, D is drought treatment. Soil-WC, Soil water content; SOC, Soil organic carbon; TN, Soil total nitrogen; AP, Soil available phosphorus; S-CAT, Soil catalase; S-ACP, Soil acid phosphatase; S-UE, Soil urease; S-ACPT, Soil acid protease; *Fv/Fm*, Chlorophyll fluorescence parameter; MDA, malondialdehyde; Pro, proline. **(C)** Correlation analysis between α diversity index and environmental factors (X = non-significant at *P* < 0.05).

Chao 1 and Shannon indices revealed the changes in microbial richness and diversity in sugarcane rhizosphere under different treatments (Figures 1A,B). There was no significant difference in the Chao 1 index among the six treatments (*F* = 1.554, *P* = 0.251). However, Shannon index revealed a higher bacterial diversity in the rhizosphere of GT21 and GT31 than in that of GT42, and there were significant differences between GT21, GT31, and GT42 under drought conditions (*F* = 8.521, *P* = 0.005). Shannon index was positively correlated with AP and S-ACP levels (*P* < 0.05) and negatively correlated with S-ACPT levels, whereas Chao1 index was positively correlated with AP levels and leaf water potential (*P* < 0.05) and negatively correlated with S-ACPT levels (*P* < 0.05) (Figure 1C and Supplementary Figure 2). The combined effect of water treatment and cultivar had a significant effect on AP levels (*P* < 0.05) and extremely significant effect on S-CAT levels (*P* < 0.001). SOC, TN, and S-ACP levels were significantly different among the treatments (*P* < 0.001). For the same sugarcane variety, drought treatment had extremely significant effects on soil nutrients and enzyme activities (*P* < 0.001) (Table 1).

**TABLE 1:** Soil physical and chemical properties were significantly different under different treatments.

Water	Varieties	Leaf water potential	Fv/Fm	MDA	Pro
Drought	GT42D	−1.2187a	0.681a	15.5633de	57.3933b
	GT21D	−0.819c	0.7333b	14.52d	225.4467e
	GT31D	−0.983b	0.7243b	16.7333e	131.46d
Control	GT42C	−0.2313d	0.7873c	6.78a	49.1867ab
	GT21C	−0.112d	0.7933c	10.02c	38.5a
	GT31C	−0.199d	0.783c	8.5233b	104.5733c
Water		<0.001***	<0.001***	<0.001***	<0.001***
Varieties		<0.001***	<0.001***	<0.001***	<0.001***
Water × Varieties		<0.001***	<0.001***	<0.001***	<0.001***

^a^Values are mean of three soil samples. Soil-WC, Soil water content; SOC, soil organic carbon; TN, total nitrogen; AP, Available phosphorous; S-CAT, Soil catalase; S-ACP, Solid-acid phosphatase; S-UE, Soil Urease; S-ACPT, Solid -Acid Protease. ^b^Different letters indicate significant differences (ANOVA, P < 0.05, Turkey’s HSD post hoc analysis) among differences treatments. ***P value < 0.001.

**FIGURE 2:**
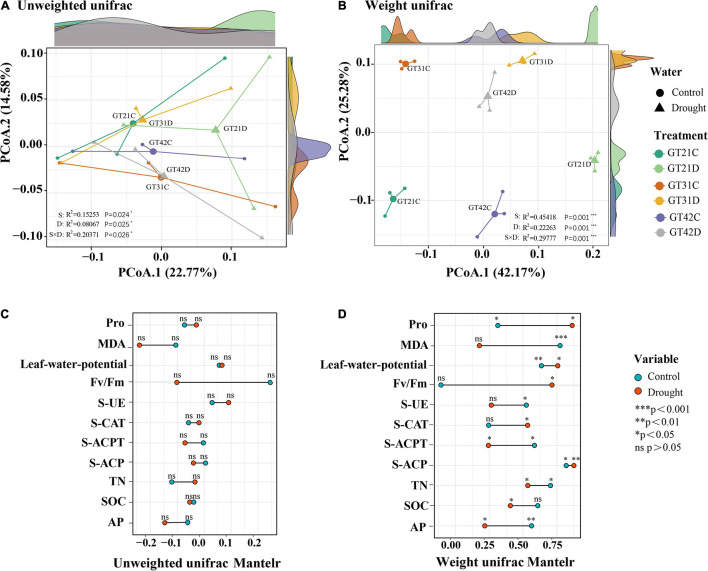
Beta diversity analysis revealed similarities in bacterial community composition between different treatments. The principal coordinate analysis based on unweighted Unifrac distance **(A)** and the principal coordinate analysis based on weighted Unifrac distance **(B)** for bacterial community composition among different treatments. Axis.1 represents the contribution rate of water treatment to differences among treatments, and Axis.2 represents the contribution rate of varieties to differences among treatments. Correlation between environmental factors and phylogenetic members and composition of sugarcane was analyzed using the Mantel Test **(C)** UnifracMantelr, **(D)** Weight UnifracMantel. *p < 0.05; **p < 0.01; and ***p < 0.001.

The unweighted UniFrac distance and weighted UniFrac distance were used to visualize the separation mode of rhizosphere bacteria under different treatments (Figure 2). Without considering species abundance, the distribution of community composition among treatments was relatively concentrated, and the effects of drought and variety difference on bacterial community composition were 22.77 and 14.58%, respectively (Figure 2A). According to the weighted Unifrac analysis, the bacterial community composition was scattered among all treatments considering species abundance, and there was no overlapping area. The influence of drought on the bacterial community composition was 42.17%, while the influence of variety difference was only 25.28% (Figure 2B). The Mantel test results showed that the β-diversity of rhizosphere bacteria under drought stress was correlated with physicochemical properties of the soil and physiological state of sugarcane (Figures 2C,D). In the control treatment, β-diversity was significantly correlated with MDA and Pro levels, leaf water potential, and AP levels (*P* < 0.05) (Figure 2D).

### Effects of Drought Stress on Physiological Status and Rhizosphere Bacterial Community Structure of Sugarcane

The leaf water potential of the three cultivars under drought stress was significantly different (*P* < 0.05); the lowest was detected in GT42D and the highest in GT21D. There was no significant difference in the *F*_v_/*F*_m_ value of the three varieties of sugarcane in the control. Under drought stress, the *F*_v_/*F*_m_ value of GT42D was significantly lower than those of GT21D and GT31D (*P* < 0.05). There were significant differences in the MDA and Pro content of sugarcane among the treatments (*P* < 0.001). The difference in MDA content between GT42D and GT42C was the largest, and the highest MDA content was detected in GT21D. Compared with that of control group, the Pro content of GT21D increased most significantly and the Pro content of GT42D changed the least under drought stress (Table 2).

**TABLE 2:** The physiological status of sugarcane under different treatments was significantly different.

Water	Varieties	Soil-WC	SOC	TN	AP	S-CAT	S-ACP	S-UE	S-ACPT
Drought	GT42D	0.067a	12.5668bc	0.8273d	21.2903a	20.6346c	1.2501b	1.3975a	1.8448cd
	GT21D	0.0778a	8.8961a	0.3475a	21.4023a	15.3594b	2.0069e	1.6354ab	1.8108c
	GT31D	0.0804a	21.2769d	1.2105e	22.2885bc	22.2789d	1.6222d	1.4675ab	1.1647b
Control	GT42C	0.1519b	13.5975c	0.8092d	21.577ab	21.609d	1.0536a	1.6839b	1.9462d
	GT21C	0.1564b	10.1564a	0.6744c	22.6376c	21.886d	1.4174c	2.1689c	1.0202a
	GT31C	0.1661b	10.5002ab	0.5371b	22.5682c	13.5786a	1.2543b	1.4078a	1.8108c
Water		0.04939*	<0.001***	<0.001***	<0.001***	<0.001***	<0.001***	<0.001***	<0.001***
Varieties		<0.001***	<0.001***	<0.001***	<0.001***	0.0138*	<0.001***	<0.001***	0.4787
Water × Varieties		0.7417	<0.001***	<0.001***	0.0204*	<0.001***	<0.001***	<0.001***	<0.001***

^a^Values are mean of three soil samples. Fv/Fm, Chlorophyll fluorescence parameter; MDA, Malondialdehyde; Pro, Proline. ^b^Different letters indicate significant differences (ANOVA, P < 0.05, Turkey’s HSD post hoc analysis) among differences treatments. ^c^*0.01 < P value < 0.05; ***P value < 0.001.

**FIGURE 3:**
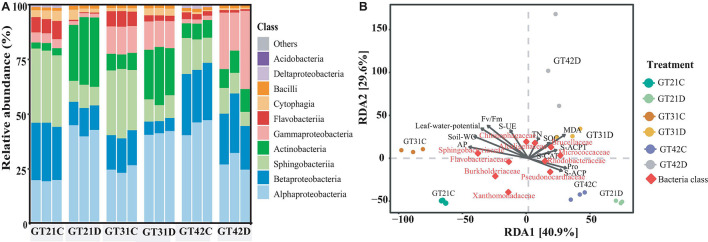
The relative abundance of bacterial species in the rhizosphere bacterial community and the dbRDA redundancy analysis with environmental factors. **(A)** Relative abundance of dominant rhizosphere flora under different treatments, with three repetitions in each treatment (Top 10 in terms of OTUs). **(B)** Distance-based redundancy analysis between different varieties, water treatments, and environmental factors and dominant flora.

The 16S rDNA analysis of rhizosphere soil bacteria showed that the composition of rhizosphere bacteria of different sugarcane varieties was similar, but under drought treatment, they showed certain differences among sugarcane varieties (Figure 3A). Alphaproteobacteria, Gammaproteobacteria, Actinobacteria, Sphingobacteriia, and Betaproteobacteria were the relatively abundant basic bacteria in the rhizosphere flora of sugarcane. Compared with that in GT21C, the relative abundance of Actinobacteria and Alphaproteobacteria in the rhizosphere bacterial community of GT21D increased, while the relative abundance of Betaproteobacteria and Sphingobacteria decreased. The change in bacterial relative abundance of GT31 under drought treatment is similar to that of GT21, while the change of bacterial composition in the rhizosphere of GT42 is different from that of GT21 and GT31. Compared with that in GT42C, the relative abundance of Alphaproteobacteria in GT42D decreased, and the relative abundance of Gammaproteobacteria increased significantly (Figure 3A). From the Venn diagram of rhizosphere bacteria species, it can be seen that the shared rhizosphere bacteria species of the three varieties under drought treatment are not significantly different from those under normal treatment (Supplementary Figure 3).

According to the dbRDA results, drought stress and cultivar type explained the differences in the composition of sugarcane rhizosphere bacteria at 40.9 and 29.6%, respectively (Figure 3B). The distribution of GT42D rhizosphere flora OTU was relatively dispersed; GT42D and GT42C flora OTUs were distributed on both sides of the RDA2 axis and were significantly affected by drought. The bacterial community OTUs of the control and drought treatments of GT21 and GT31 were distributed on both sides of the RDA1 axis (Figure 3B and Table 3). The red square represented by Rhodobacteraceae, Brucellaceae, and Pseudonocardiaceae abundance overlapped the area of the soil enzymatic activity and the sugarcane Pro content. The red square of Sphingobacteriaceae and Flavobacteriaceae abundance were close to the arrow points of AP levels (Figure 3B).

**TABLE 3:** Correlation display of each factor and principal component of RDA diagram.

Indicators	*R* ^2^	Pr(>*r*)
Soil_WC	0.235641	0.006**
SOC	0.275288	0.003**
TN	0.38945	0.001***
AP	0.311788	0.002**
S-CAT	0.288824	0.002**
S-ACP	0.292114	0.004**
S-UE	0.467151	0.001***
S-ACPT	0.422553	0.002**
Leaf-water-potential	0.319814	0.001***
Leaf-photosynthesis	0.319965	0.001***
Fv/Fm	0.257374	0.005**
MDA	0.216719	0.01**
Pro	0.367849	0.001***

*^a^The R^2^ of the environmental factor of the RDA chart and the significance p-value.*

^b^**P value < 0.01; ***P value < 0.001.

### Main Response Strains of Sugarcane Rhizosphere Bacteria Under Drought Stress

**FIGURE 4:**
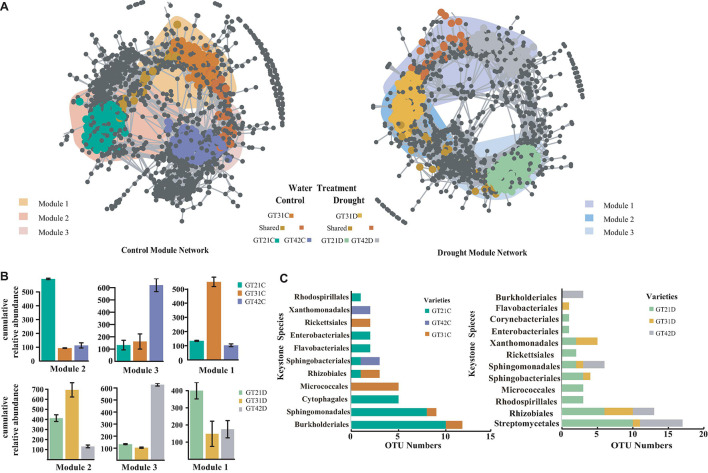
Co-occurrence pattern of sugarcane sensitive OTUs. **(A)** Co-occurrence network showed the connectivity between the core rhizosphere flora OTUs of each sugarcane variety under water and drought treatment (ρ > 0.7, *P* < 0.001; Indicated with gray lines). Gray nodes represent OTUs that are insensitive to variety differences; areas with different colors represent the aggregation modules of dominant rhizosphere flora of different varieties; nodes with different colors correspond to the key dominant flora of different varieties. The lines between the nodes represent the correlation between the connected OTU. **(B)** The cumulative relative abundance of bacterial OTUs in the aggregation module of different sugarcane varieties under water and drought treatment. **(C)** Display Keystone Species of different Species according to the number of OTU in the cOTU co-occurrence and abundance of Keystone (Supplementary Figure 4).

The co-occurrence network analysis was used to visualize the symbiotic relationships among the rhizosphere bacterial OTUs of the three cultivars under different water treatments (Figure 4). The rhizosphere response bacteria of different sugarcane cultivars were primarily concentrated in different modules (M1, M2, and M3). Under the control treatment, the three modules exhibited a high degree of aggregation and a large overlap area. Under drought stress, the different modules were separated, and the number of shared OTUs was reduced (Figure 4A). The co-occurrence and richness analysis of the core flora csOTUs (cropping sensitive OTUs) determined the bacterial keystone species under different treatments (Supplementary Figure 4). In the control, M2 was dominated by the core flora of GT21C, while M3 and M1 corresponded to GT42C and GT31C, respectively. Under drought stress, the cultivars with higher cumulative relative abundance of bacterial OTUs in M2 and M1 were GT31 and GT21, and GT42 rhizosphere bacterial OTUs were dominant in M3 (Figure 4B). Sphingomonadales were the rhizosphere core bacteria of GT21C; Burkholderiales, Xanthomonadales, and Sphingobacteriales comprised the core response bacteria of GT42C, and Micrococcales was the rhizosphere core bacteria of GT31C. Under drought stress, the main flora of GT21D included Streptomycetales and Rhizobiales; the main core bacteria of GT42D were Burkholderiales, Sphingomonadales, Rhizobiales, and Streptomycetales; and the core species of GT31D were Flavobacteriales, Xanthomonadales, and Rhizobiales (Figure 4C). Under the control treatment, Burkholderiaceae was the core species shared by the three sugarcane varieties. The relative abundance of Burkholderiaceae in the rhizosphere communities of GT31D and GT21D decreased, and the abundance of Brucellaceae and Xanthomonadaceae increased in the treated GT42D. Although Streptomycetaceae abundance increased in GT21D and GT31D under drought stress, there was no significant change in GT42D (Supplementary Figure 5). Further analysis of bacterial species revealed that Lechevalieria was relatively abundant only in GT21D, while Ochrobactrum was relatively abundant only in GT42D. While Chitinophaga showed high relative abundance in both GT21D and GT21C (Supplementary Figure 6).

Correlation analysis between core responding bacteria and environmental factors showed that AP levels, S-ACP levels, S-UE levels, Pro content, and *F*_v_/*F*_m_ values were related to core responding bacteria (Supplementary Figure 7). There was a significant positive correlation between abundance of Streptomycetales and Pseudonocardiales, and Pro content (*P* < 0.001). S-UE level was significantly negatively correlated with abundance of Rhizobiales and Micrococcales (*P* < 0.001) but significantly positively correlated with abundance of Burkholderiales (*P* < 0.001). Flavobacteriales abundance had a very significant positive correlation with soil water content, AP level, and *F*_v_/*F*_m_ value (*P* < 0.001), and a significant correlation with leaf water potential (*P* < 0.01) (Supplementary Figure 7).

### Structural Equation Model Determines the Correlation Path Among Variables

**FIGURE 5:**
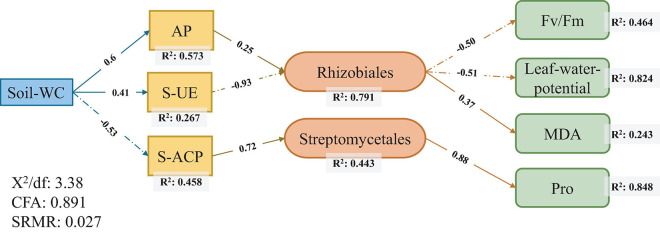
Structural equation modeling of the relationship between core responsive bacteria and soil environment and sugarcane physiology under drought stress.

Structural equation model was used to test the relationship of sugarcane rhizosphere drought-response strains with soil conditions and sugarcane physiological conditions and to explore the ways in which soil and plants affect rhizosphere core response bacteria. The results showed that the soil moisture content most directly affect the physical and chemical properties of the soil. Among them, the AP level (*R*^2^ = 0.573) had the strongest correlation with soil water content, followed by S-ACP (*R*^2^ = 0.458) and S-UE (*R*^2^ = 0.267) levels. Among the sugarcane plant physiological indicators, there was a high correlation between *F*_v_/*F*_m_ values (*R*^2^ = 0.464), leaf water potential (*R*^2^ = 0.824), MDA levels (*R*^2^ = 0.243), and Pro content (*R*^2^ = 0.848) (Figure 5).

## Discussion

### Drought Stress Affected Bacterial Diversity in Sugarcane Rhizosphere

Plants gradually shape the root microorganisms they need in the long evolutionary process, which in turn affect the host plant’s response to biological and non-biological environmental stress factors (Fitzpatrick et al., 2018). In this experiment, we found that there was no significant difference in the diversity of sugarcane rhizosphere bacteria under normal water condition, but there was a significant change under drought stress, and these differences were correlated with varieties and environmental conditions (Figures 1, 2). The study of Hakim et al. (2021) also showed that the stable ecological structure of rhizosphere microbial community formed in plant rhizosphere changes in accordance with changes in the physicochemical properties of rhizosphere soil and plant growth status. In Chao1 and unweighted Unifrac analyses, there was no significant change in bacterial diversity among the treatments, indicating that the sugarcane rhizosphere bacterial diversity has a certain degree of stability (Figures 1A, 2A). Through Pearson correlation and Mantel analyses, we found that the correlation between β-diversity of rhizosphere bacteria and MDA content is extremely significant (*P* < 0.01), and the correlation between Pro content and leaf water potential, S-UE levels, S-ACPT levels, S-ACP levels, TN levels, and AP levels all showed significant correlation (0.01 < *P* < 0.05, Figures 1C,D).

Under drought stress, plants can resist the damage caused by adverse conditions by adjusting the content of MDA and Pro in the body, and this process is accompanied by changes in root exudates, which affects the bacterial components in the rhizosphere environment (Timmusk and Wagner, 1999; Mayak et al., 2004). Moreover, soil moisture conditions directly affect the existence and activity of soil enzymes. Many studies have shown that the abundance of soil microorganisms is positively correlated with enzyme activity (Dinesh et al., 2012). However, Zeng et al. (2014) believed that changes in physical and chemical characteristics of the soil would lead to changes in levels of soil C, N, P, and other nutrients, thus increasing the abundance of bacteria involved in material circulation and changing the structure of rhizosphere bacteria. Rhizosphere bacteria affect the stress resistance of host plants by regulating soil nutrient conversion and promoting root nutrient acquisition (Dobbelaere et al., 2003). The results of our experiment showed that the changes in rhizosphere bacterial diversity under drought stress were correlated with physical and chemical properties of the soil, and was also significantly correlated with AP and S-ACP levels (Figure 1C and Supplementary Figure 2). Available phosphorous in soil that can be absorbed by plants originates mostly from phosphate anions (Arcand and Schneider, 2006). Under drought stress, the mobility and conversion of cured P level in soil decreases, leading to a decrease in the concentration of phosphate ions in the soil solution, thereby affecting the AP content of the rhizosphere soil (Richardson, 2001). Moreover, soil microorganisms can mobilize S-ACP, accelerate the degradation of organic P substrates, and promote the generation of phosphate to supply AP needed by plants (Gargallo et al., 2018). These results indicate that drought stress can break the original equilibrium state of sugarcane rhizosphere bacteria and change their diversity. These changes are closely related to soil nutrient cycling in the rhizosphere soil environment.

### The Change in Rhizosphere Bacterial Community Is Related to Drought Tolerance of Sugarcane

The main families of sugarcane rhizosphere bacteria are Proteobacteria (29.6%), Acidobacteria (23.4%), Bacteroidetes (12.1%), Firmicutes (10.2%), and Actinobacteria (5.6%) (Pisa et al., 2011). The rhizosphere bacterial communities of the three sugarcane cultivars were dominated by the above bacterial classes, and the relative abundance of bacteria among the cultivars was similar under the control treatment (Figure 3). Bacteria whose relative abundance varied greatly under drought conditions and the growth-promoting bacteria mainly belonged to Actinobacteria, Proteobacteria, or Bacteroidetes (Compant et al., 2010). However, the changes in rhizosphere bacterial abundance in the three cultivars under drought stress were not consistent (Figure 3A). We attribute the differences in the collected rhizosphere flora of host plants to differences in genotypes among the three cultivars (Berendsen et al., 2012).

The differences in drought tolerance of the three sugarcane cultivars were confirmed by the broad-based drought tolerance indicators such as leaf water potential, MDA level, Pro content, and *F*_v_/*F*_mvalue_ (Ferreira et al., 2017; Chiconato et al., 2019). Among the three sugarcane cultivars, GT21 exhibited the best performance in terms of drought resistance, GT42 was the most sensitive to water, and GT31 was intermediate between the two (Table 2). Vurukonda et al. (2016) suggested that the difference in drought tolerance ability of host plants leads to different changes in rhizosphere microorganisms under drought stress. Similar responses to drought stress were observed in this study: compared with that in the control treatment, in GT21D and GT31D, the changes in the relative abundance of rhizosphere bacteria were primarily attributed to the changes in Actinobacteria abundance, whereas in GT42D, the changes were because of the changes in Proteobacteria abundance (Figure 3). However, the changes in soil flora observed under drought conditions are typically related only to the changes in drought-sensitive bacterial species, rather than the changes in the overall flora (Naylor and Coleman-Derr, 2018). Therefore, we speculated that the more stable diversity of the rhizosphere bacteria of highly drought-tolerant cultivars under drought stress was due to the increased abundance of specific bacteria in their rhizospheres. The network analysis of rhizosphere bacterial communities under different treatments revealed that the core bacterial communities of the three sugarcane varieties under drought treatment were significantly more isolated between OTU modules than under the control treatment (Figure 4). This indicated that the rhizosphere bacteria showed evident functional division under drought stress, where the drought-resistant bacteria play a role (Naylor and Coleman-Derr, 2018). Sphingomonadales, Rhizobiales, and Streptomycetales were the common core bacteria in the three sugarcane cultivars under drought stress (Supplementary Figures 5, 6). Notably, in the control treatment, no common core bacteria were found in the rhizosphere bacterial OTUs of the three cultivars, although members of Sphingomonadales, Rhizobiales, and Streptomycetales were detected among the core key bacteria of GT21C; the only key strain of GT42C was Sphingobacteriales (Figure 4C). Streptomycetales and Rhizobiales are known to promote the development of plant roots under drought conditions, and Rhizobiales are rhizosphere symbiotic bacteria that facilitate plant nitrogen fixation (Pieterse et al., 2014; Rosenberg, 2014). The Sphingomonadales class of bacteria regulates the production of plant hormones and maturation of host plants (Nakayasu et al., 2021). Owing to the function of the core flora under drought stress, nutrients and related enzymes in the rhizosphere environment are consumed (Rubin et al., 2017; Naylor and Coleman-Derr, 2018). The physiological and biochemical processes of the core drought-resistant strains were significantly correlated with SOC, AP, S-UE, S-ACP, and S-CAT levels (Supplementary Figure 7). Therefore, drought increased the abundance ratio of drought-responsive bacterial populations in the rhizosphere, thereby resulting in other differences. Drought-tolerant cultivars could enrich more drought-responsive bacteria in the rhizosphere environment.

### Response of Core Bacterial Population to Drought Stress in Sugarcane

**FIGURE 6:**
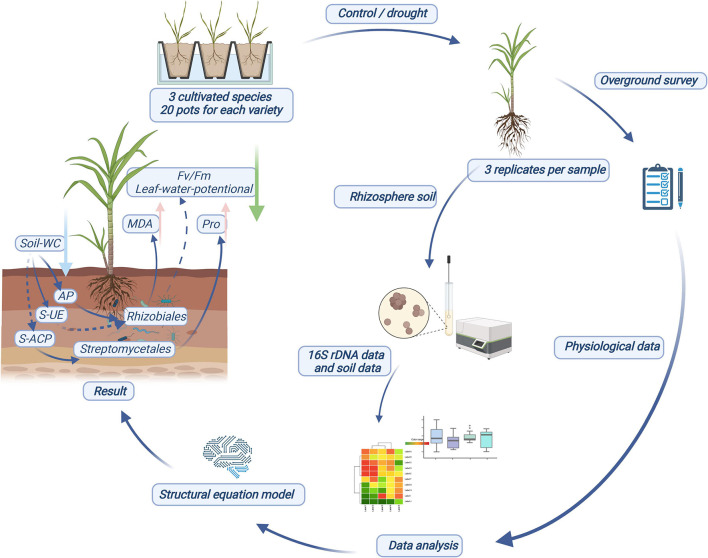
Schematic diagram of experimental design process and results.

Rhizobiales and Streptomycetales were the core responsive bacteria of the three sugarcane cultivars under drought conditions identified using SEM (Figure 5). The core responsive bacteria were significantly correlated with S-UE levels, S-ACP levels, AP levels, Pro content, leaf water potential, and *F*_v_/*F*_m_ values. Rhizobiales have been established as probiotics involved in nitrogen fixation in legumes, and recent studies have shown that Rhizobiales have an affinity for the colonization of sugarcane roots (Hart et al., 2019; Júnior I. D. A. M. et al., 2019). In our study, the abundance of rhizobia, as the core bacteria of sugarcane rhizosphere in response to drought stress and the key core strain, was correlated with S-UE and AP levels and had a direct effect on the physiological indicators of sugarcane drought resistance. The increase in AP levels can effectively alleviate the P stress caused by the imbalance of the N:P ratio in soil and promote the growth of underground plant parts (Huang et al., 2018; Hachani et al., 2020). Nitrogen-fixing nodules, an important symbiotic organ of rhizobia, provide epiphytic sites for rhizobia to convert atmospheric nitrogen to ammonia and impart a nitrogen-fixing effect to host plants (Suleiman et al., 2019; de Moura et al., 2020). In return, rhizobia obtain photosynthates from the plant. The activity of the rhizobia is affected by multiple factors such as drought resistance of the host plant, the amount of accumulated photosynthates, and levels of AP (Shen et al., 2019; Reinprecht et al., 2020). This is a good validation of the relationship between Rhizobiales and plant photosynthetic indicators in our SEM (Figure 5). Streptomycetales are plant growth promoting rhizobacteria that protect and promote plant growth by producing iron carriers, stimulating plant hormone production, and supporting phosphate solubilization (Misk and Franco, 2011; Rungin et al., 2012). S-ACP can also help in dissolving the soluble mineral phosphate in the soil, improving the availability of AP in the soil, and improving the absorption and utilization of soil nutrients by plants under stress environments (Chen et al., 2015; Mącik et al., 2020). The combined effect of Streptomycetales and S-ACP on AP level in soil may explain the direct correlation between the two. *Streptomyces* can produce bioactivators with antioxidant properties and are an important natural source of antioxidants in plants (Law et al., 2019). In addition, our results show that *Streptomyces* had a positive correlation with Pro content, and Rhizobiales had a positive correlation with MDA level. In this experiment, MDA and Pro levels were used as physiological indexes of sugarcane drought resistance. The correlations between these indexes could be regarded as the effects of two drought responsive bacteria on sugarcane drought resistance. Thus, drought-responsive rhizosphere bacteria have a positive effect on the drought adaptation of host plants through interactions with soil nutrients and enzyme activities (Figure 6).

## Conclusion

The use of sugarcane root microorganisms to manage drought stress response in plants provides a new approach for solving water scarcity problems. Our results show that the sugarcane root microflora structure is relatively stable, and drought stress causes changes in the specific response of sugarcane root microflora abundance. Drought indirectly affects the composition of rhizosphere bacterial community of sugarcane by changing soil nutrients and enzyme activities, and the changes of rhizosphere bacteria can regulate enzyme activities and photosynthesis in sugarcane and then affect the adaptability of host plants to drought. Rhizobiales and Streptomycetales are the two core bacteria playing an important role in regulating the response to drought stress in the sugarcane rhizosphere bacterial community.

## Data Availability Statement

The datasets presented in this study can be found in online repositories. The names of the repository/repositories and accession number(s) can be found below: https://www.ncbi.nlm.nih.gov/, PRJNA655948.

## Author Contributions

QL, ZW, XZ, and YL contributed to the design of the experiments, data analysis, and manuscript writing. QL, SX, and YL contributed to the experimentation. QL and ZW contributed to the data interpretation. YX and WD participated in the revision of the article. YP, JD, and BW cultivated sugarcane growth during the experiment and participated in the determination of experimental indicators. All authors contributed to the article and approved the submitted version.

## Conflict of Interest

The authors declare that the research was conducted in the absence of any commercial or financial relationships that could be construed as a potential conflict of interest.

## Publisher’s Note

All claims expressed in this article are solely those of the authors and do not necessarily represent those of their affiliated organizations, or those of the publisher, the editors and the reviewers. Any product that may be evaluated in this article, or claim that may be made by its manufacturer, is not guaranteed or endorsed by the publisher.

## Footnotes

1
http://www.usadellab.org/cms/?page=trimmomatic

2
https://ccb.jhu.edu/software/FLASH/

3
http://www.mothur.org

4
https://www.drive5.com/usearch/

5
https://www.ncbi.nlm.nih.gov/sra

## Supplementary Material

Supplementary Figure 1 Soil moisture content during sugarcane planting.

Supplementary Figure 2 Correlation of alpha-diversity index with environmental factor. Correlation of alpha-diversity index with soil properties **(A,C)** and physiological shape **(B,D)** of sugarcane (Chao 1 ≥ 0; Shannon ≥ 0).

Supplementary Figure 3 OTUs proportional Venn diagram of sugarcane rhizosphere under different treatments.

Supplementary Figure 4 Co-occurrence and abundance of dominant bacterium cOTUs in sugarcane rhizosphere. The relative abundances (as counts per million, CPM) of all sensitive OUT in the symbiosis network of rhizosphere microbiome (Figure 4A) were plotted as A function of their coexistence degree, under water treatment **(A)** and under drought treatment **(B)**. The different colored areas represent the topological roles in the entire co-occurrence network, and the nodes are colored according to the corresponding modules. The lavender region (with the highest co-occurrence of 1%) is the Keystone region with the highest degree of central connection.

Supplementary Figure 5 The relative abundance of biomarkers at the family level of rhizosphere bacteria.

Supplementary Figure 6 The relative abundance of biomarkers at the genus level of rhizosphere bacteria.

Supplementary Figure 7 Correlation analysis of main response flora and environmental factors. **p <* 0.05; ***p <* 0.01; and ****p <* 0.001.
