# Supplementary figures and images for: Response of Sugarcane Rhizosphere Bacterial Community to Drought Stress
Source: Front Microbiol. 2021 Oct 6;12:716196. doi: 10.3389/fmicb.2021.716196 (PMC8527094; doi:10.3389/fmicb.2021.716196)

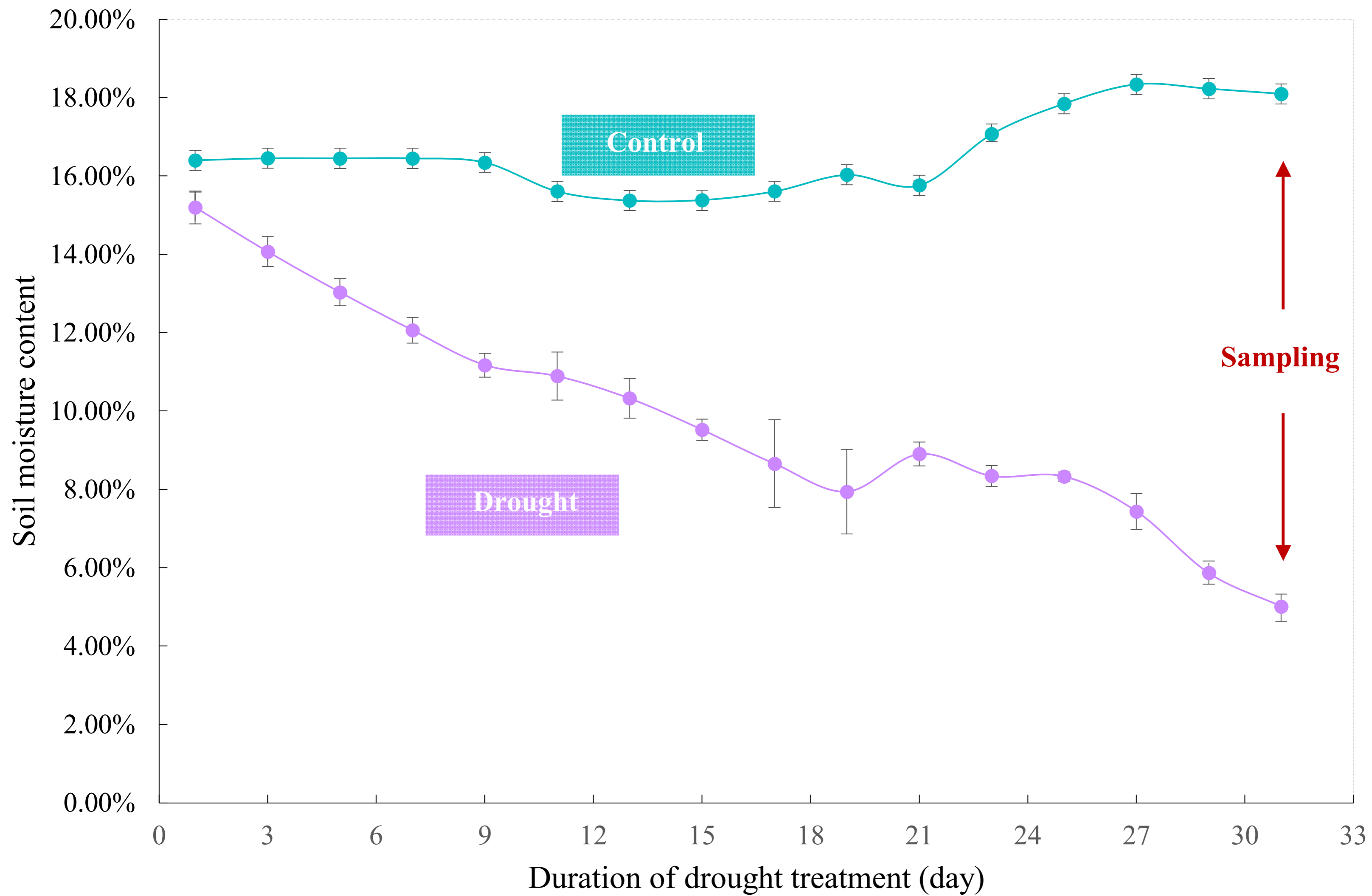

Supplement: Supplementary Figure 1 — Soil moisture content during sugarcane planting. [file Data_Sheet_1.zip › Figure 1.PDF]

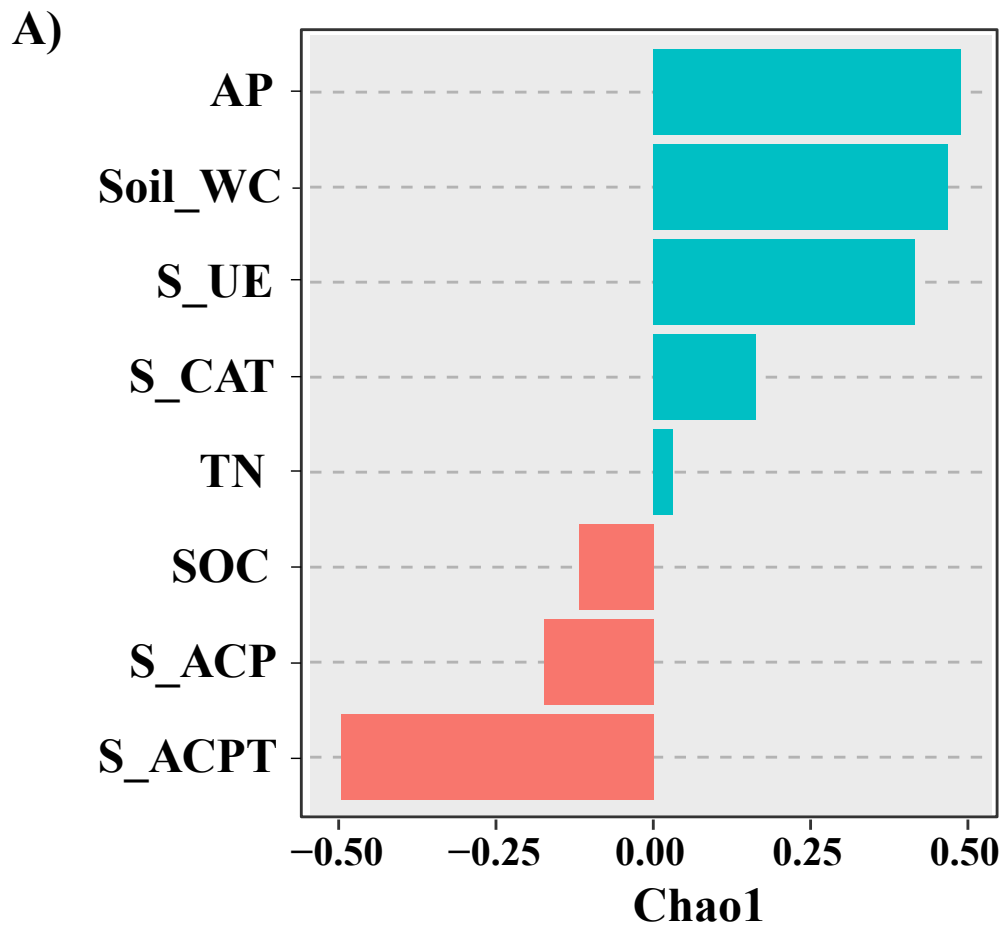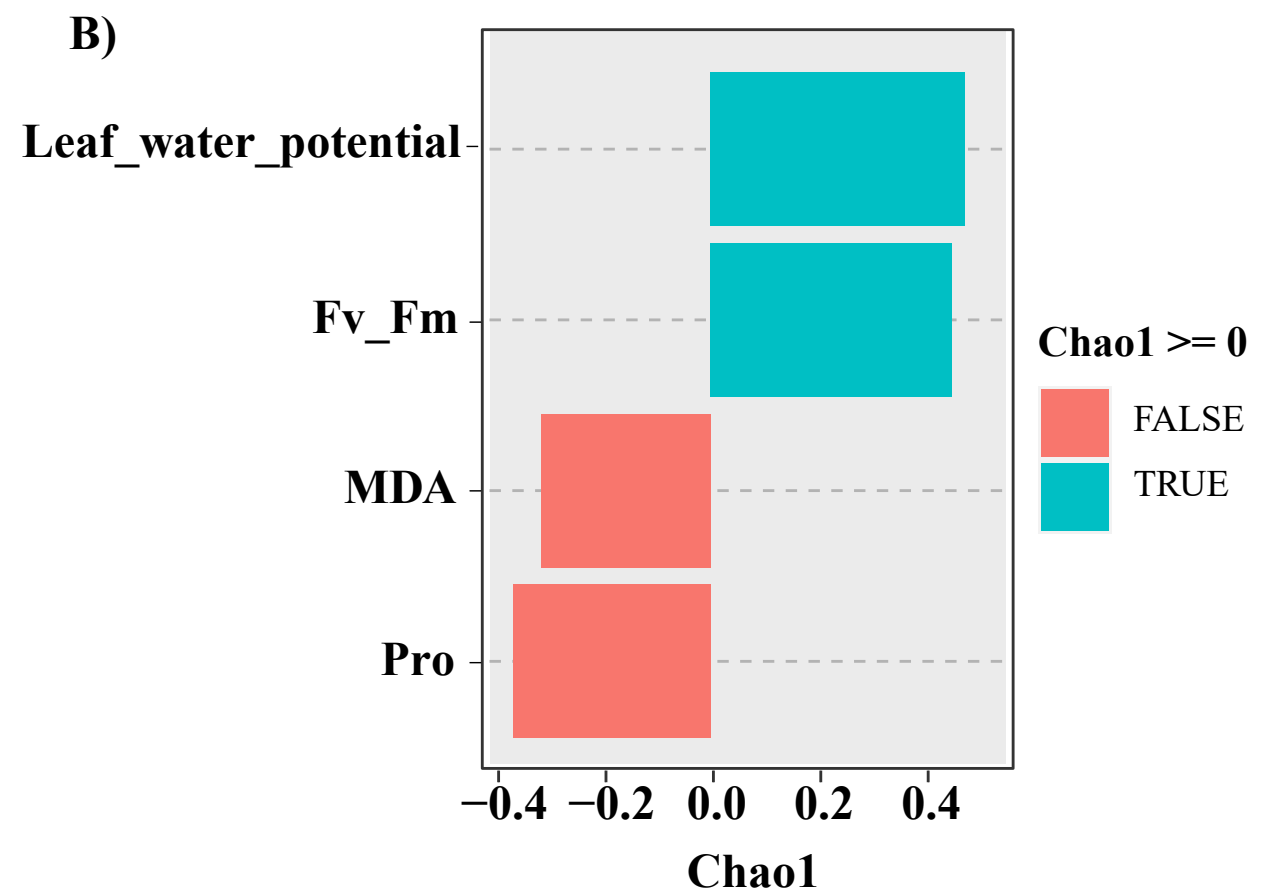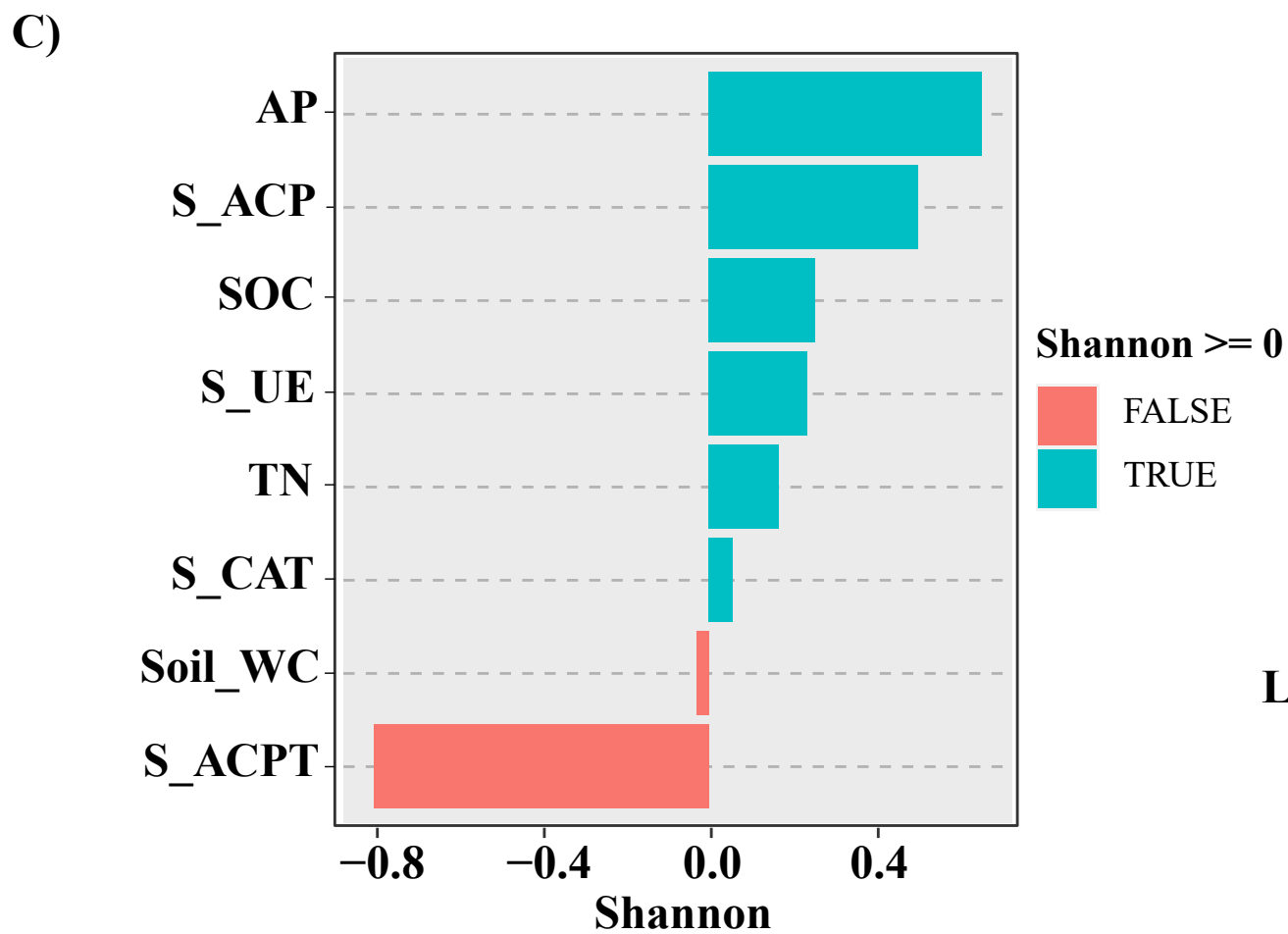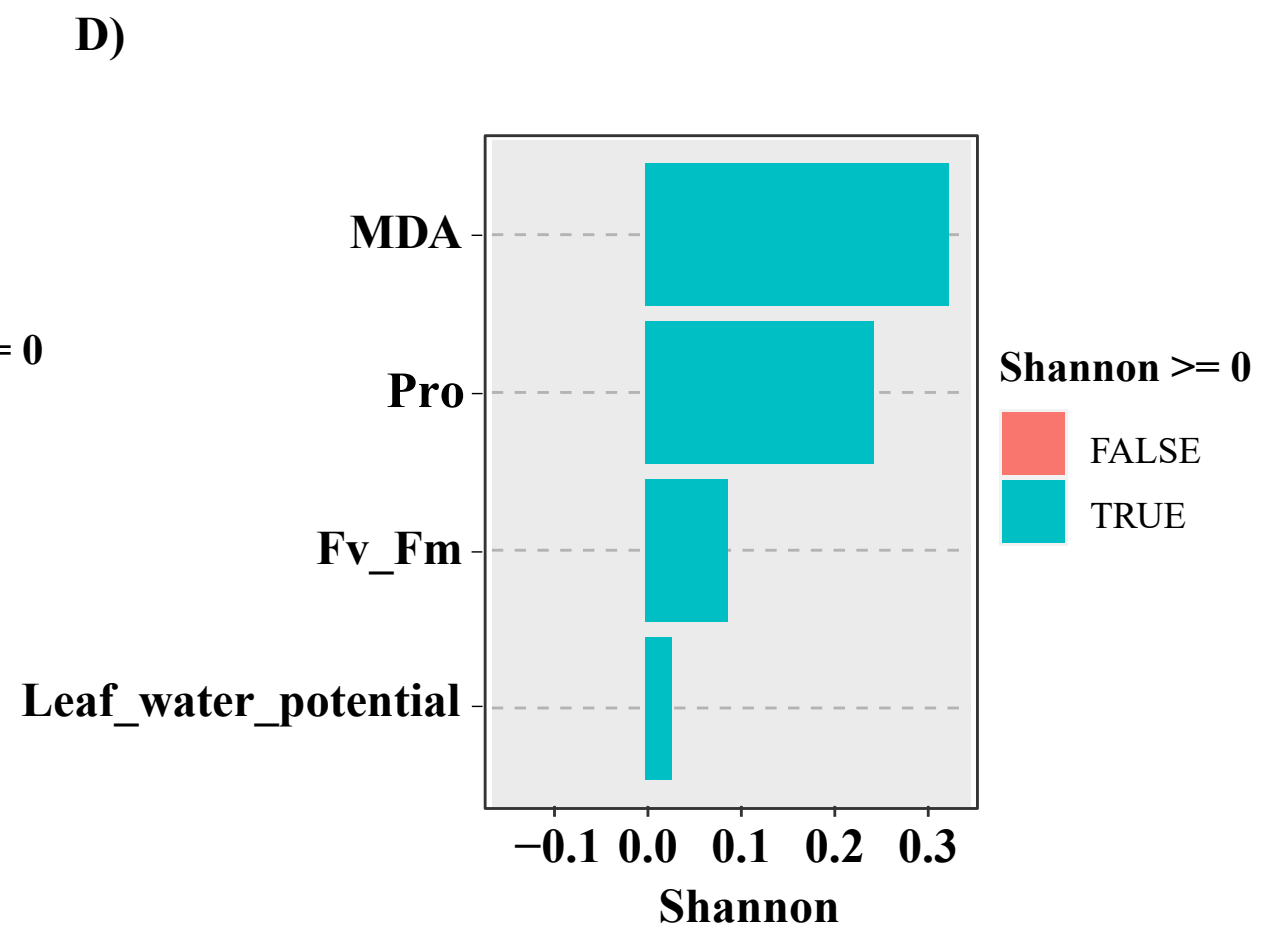

Supplement: Supplementary Figure 1 — Soil moisture content during sugarcane planting. [file Data_Sheet_1.zip › Figure 2.PDF]

**A) Control**

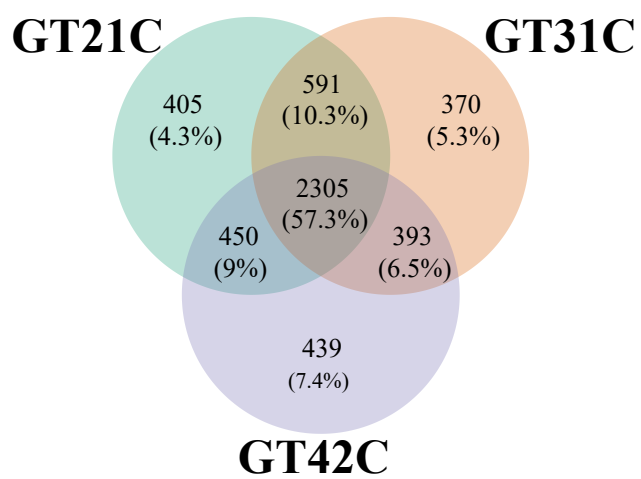

**B) Drought**

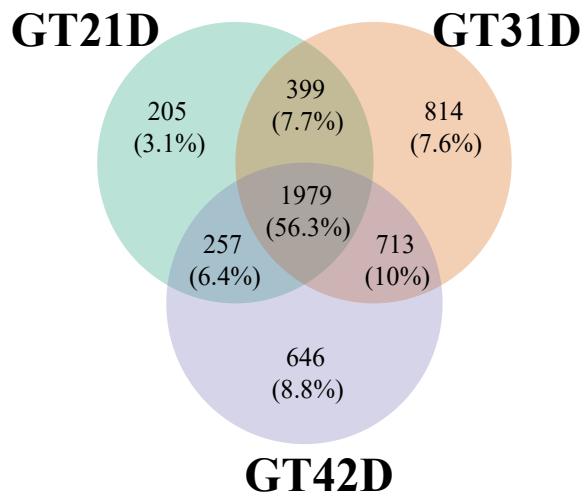

Supplement: Supplementary Figure 1 — Soil moisture content during sugarcane planting. [file Data_Sheet_1.zip › Figure 3.PDF]

A)

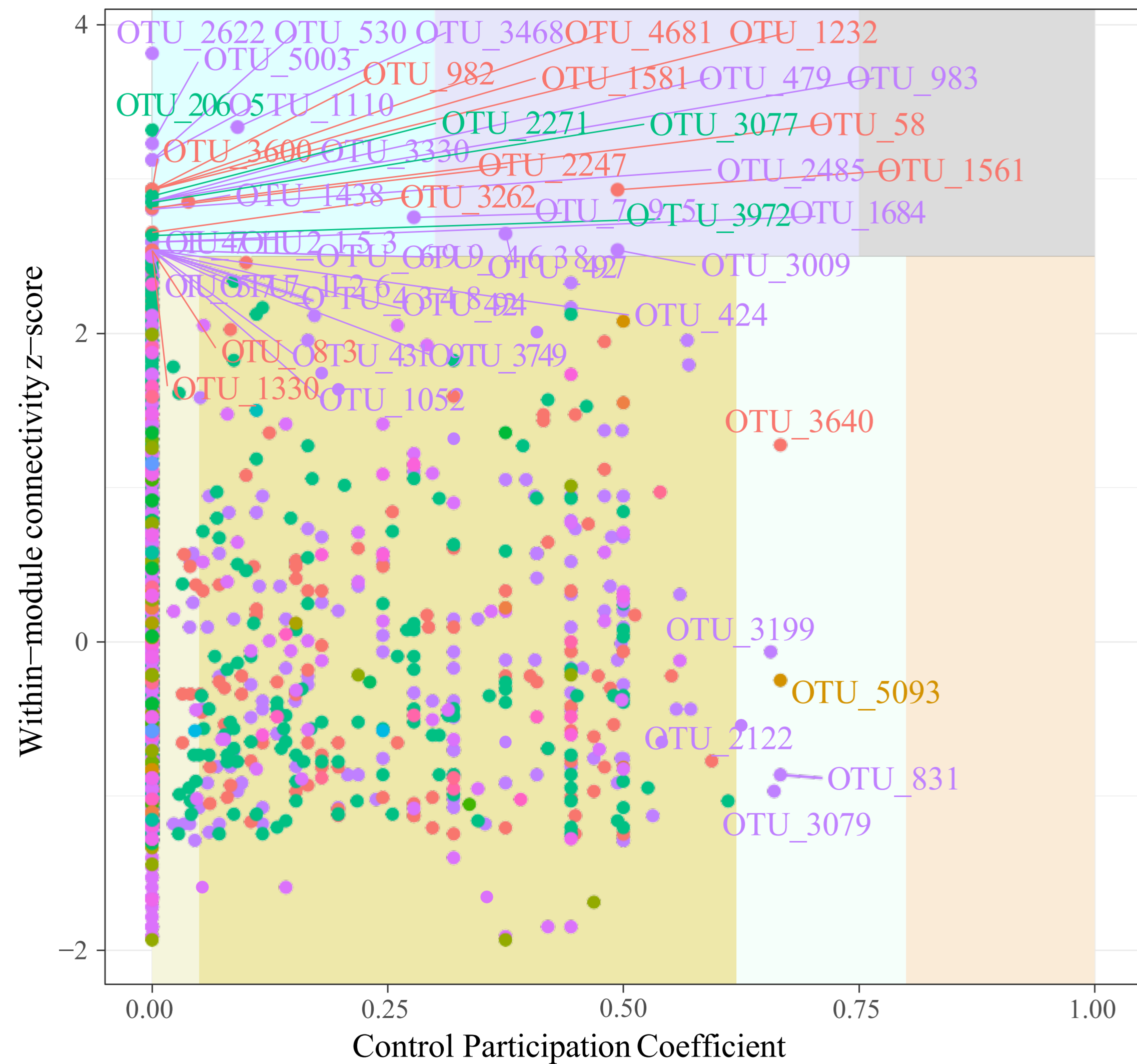

B)

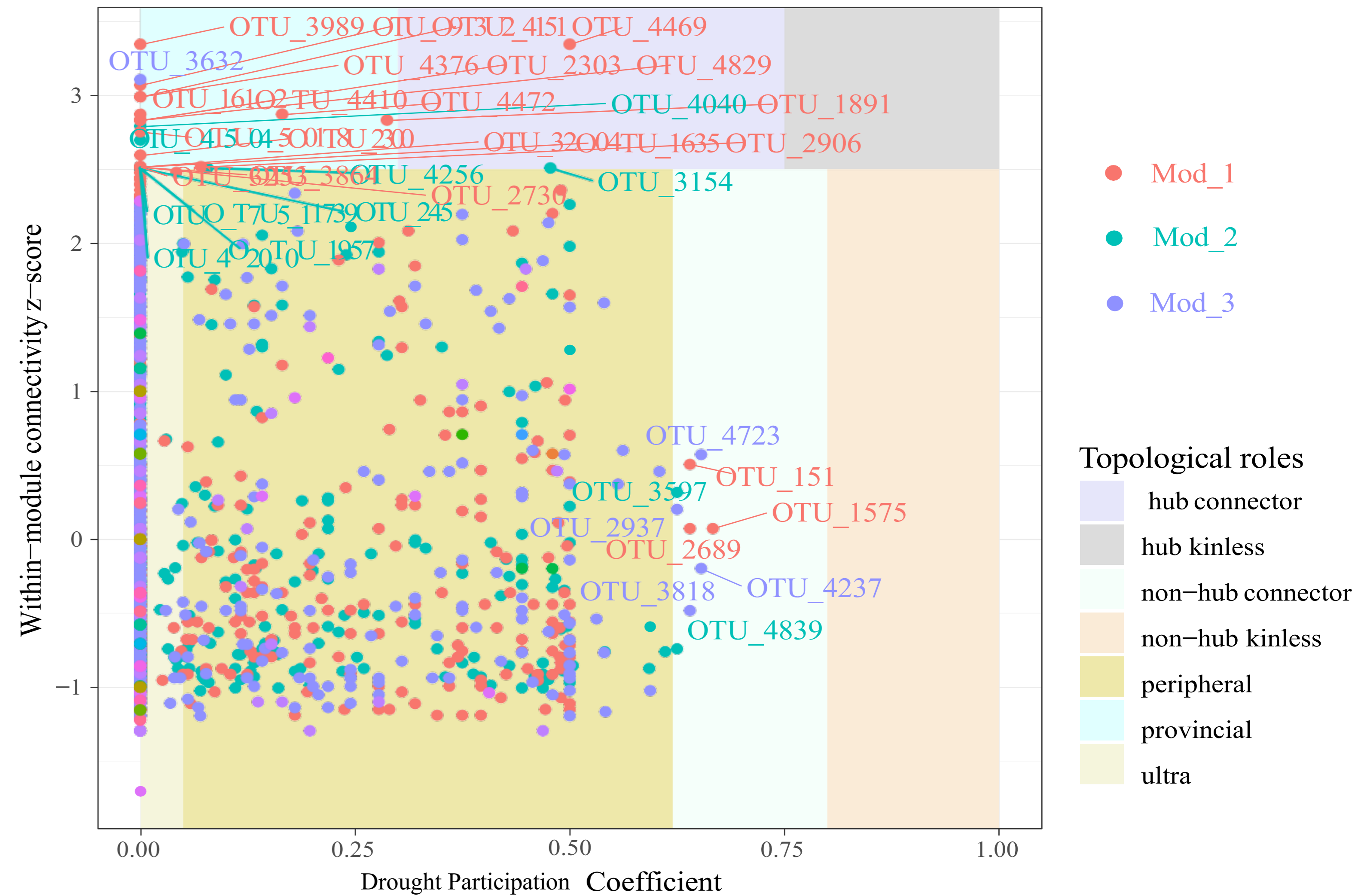

Supplement: Supplementary Figure 1 — Soil moisture content during sugarcane planting. [file Data_Sheet_1.zip › Figure 4.PDF]

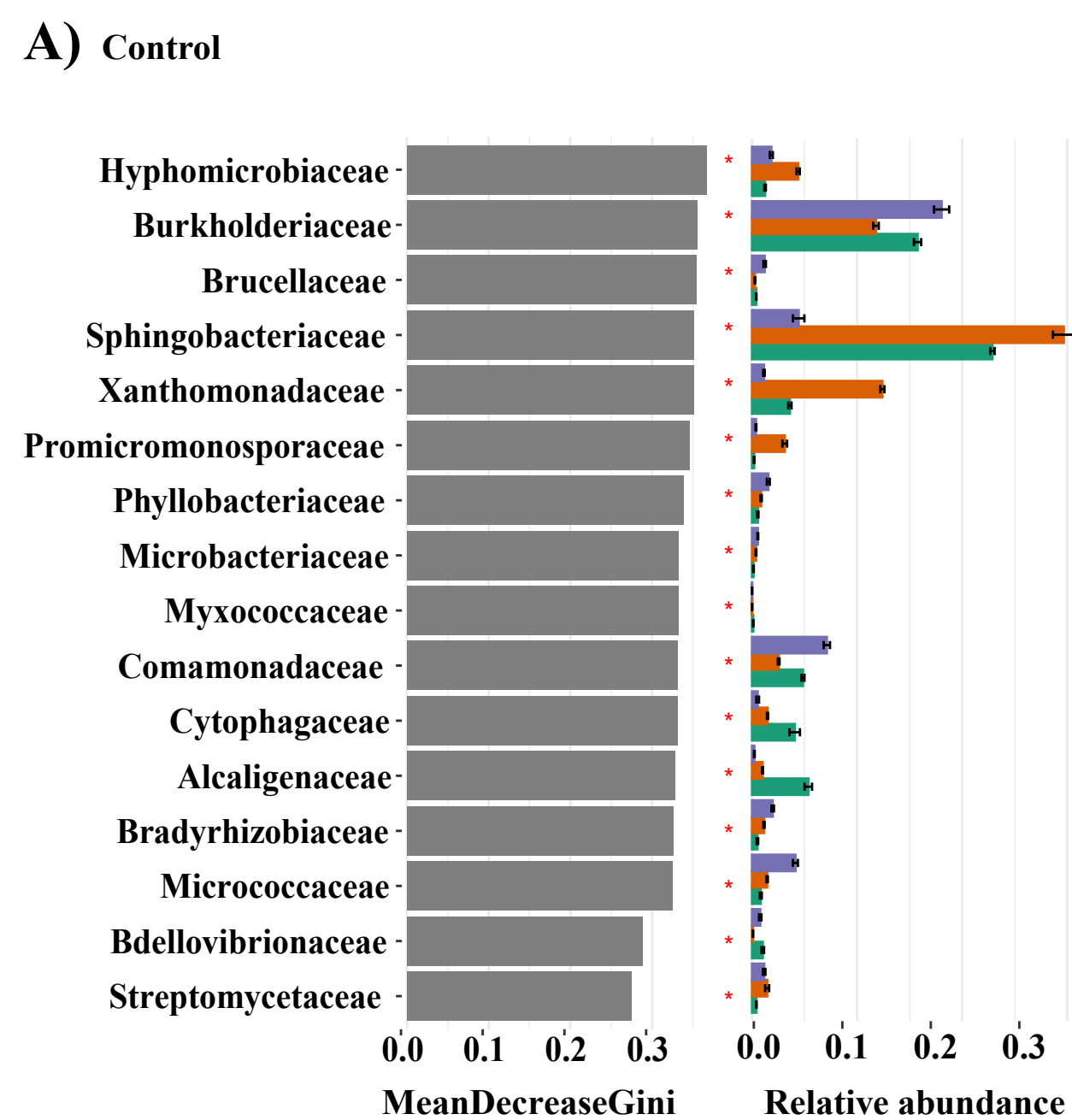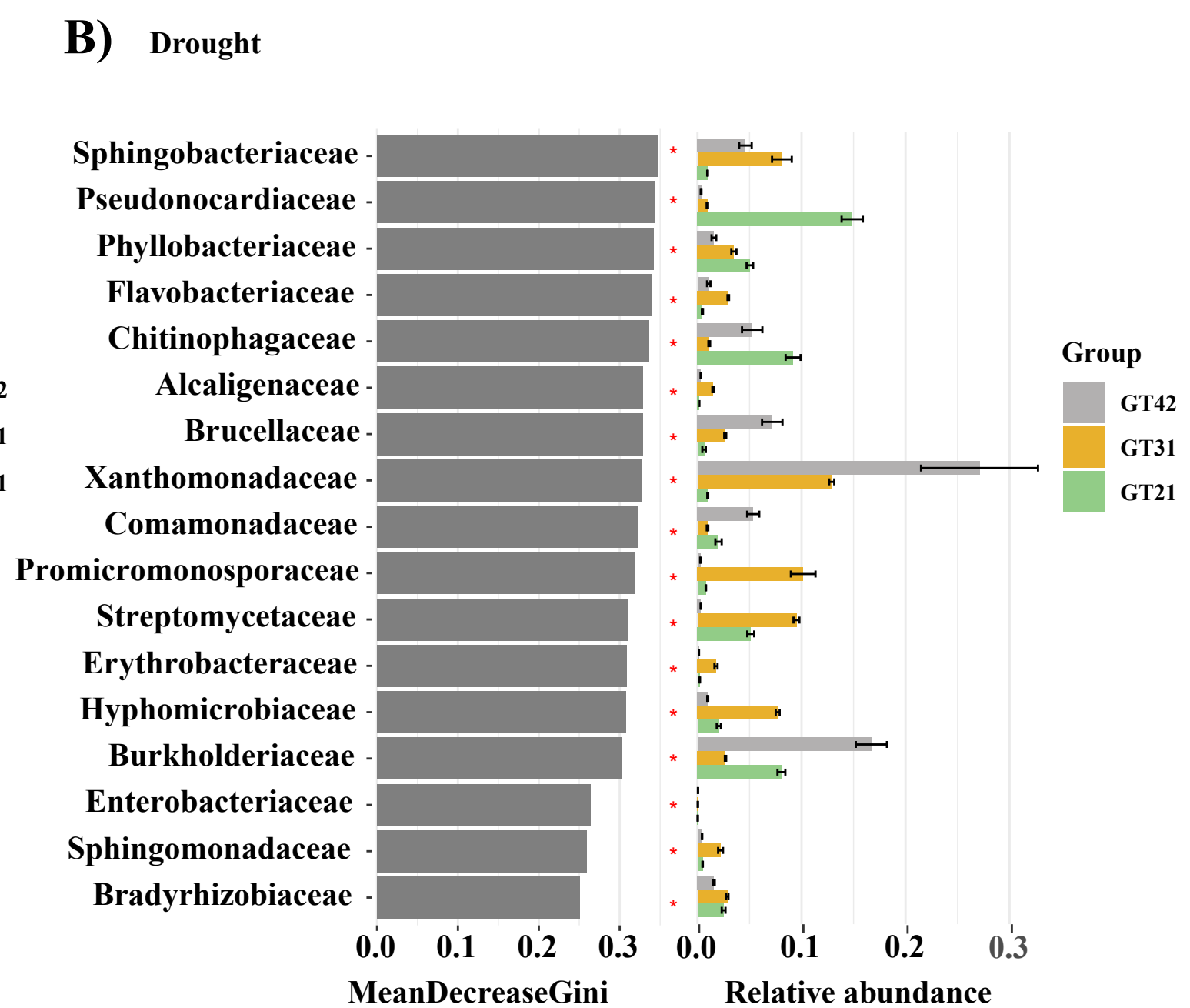

Supplement: Supplementary Figure 1 — Soil moisture content during sugarcane planting. [file Data_Sheet_1.zip › Figure 5.PDF]

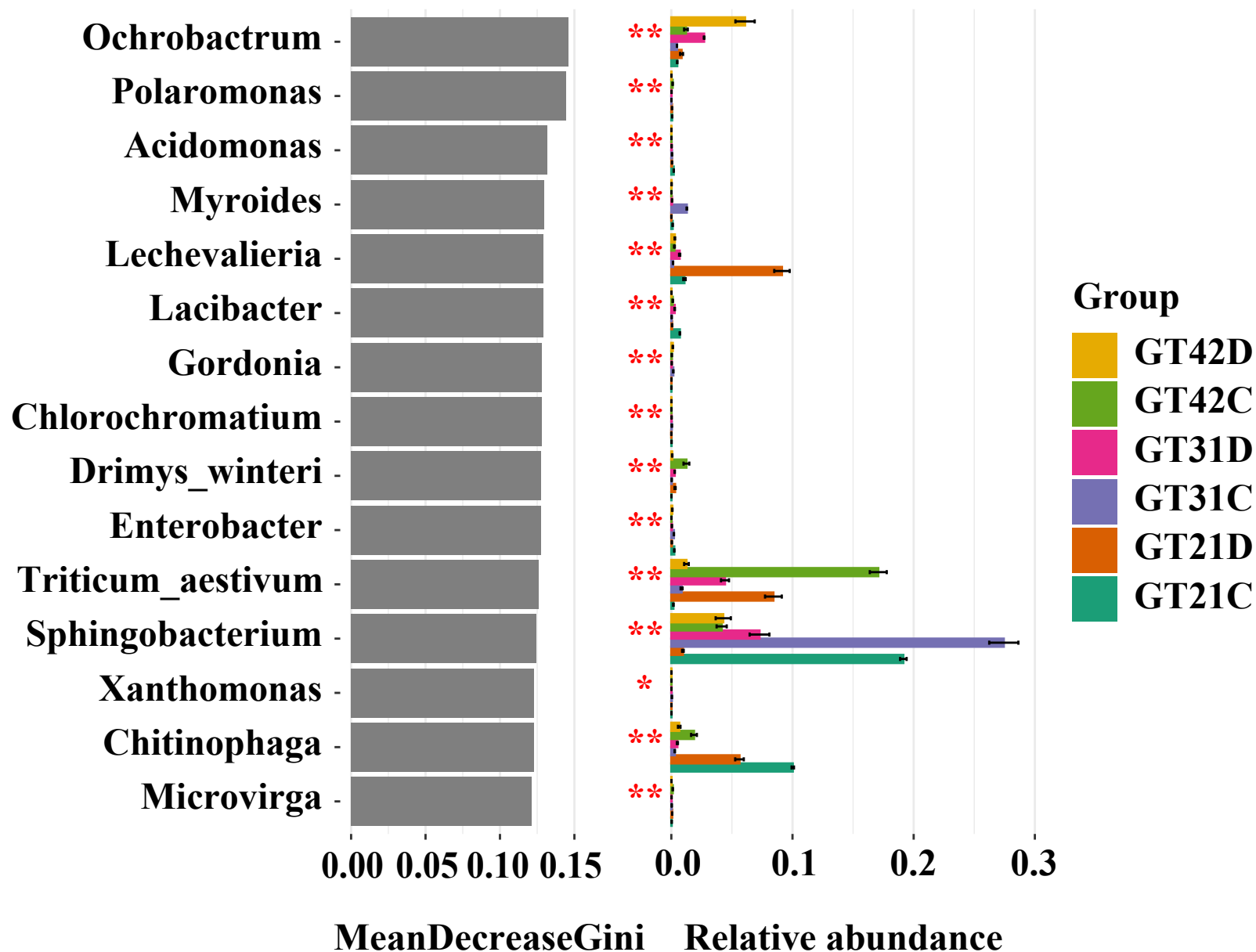

Supplement: Supplementary Figure 1 — Soil moisture content during sugarcane planting. [file Data_Sheet_1.zip › Figure 6.PDF]

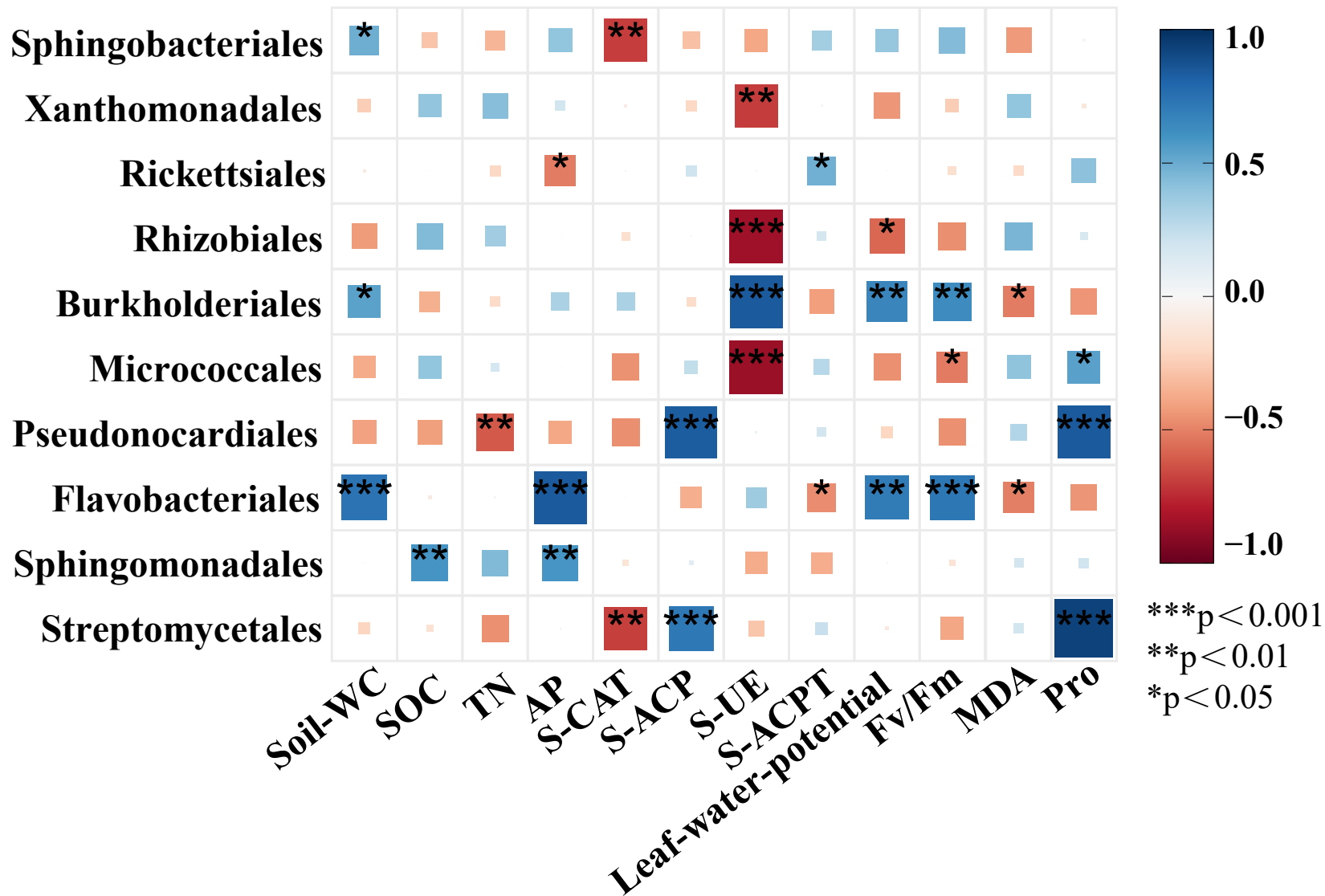

Supplement: Supplementary Figure 1 — Soil moisture content during sugarcane planting. [file Data_Sheet_1.zip › Figure 7.PDF]
